# Fitness Costs of Synonymous Mutations in the *rpsT* Gene Can Be Compensated by Restoring mRNA Base Pairing

**DOI:** 10.1371/journal.pone.0063373

**Published:** 2013-05-15

**Authors:** Peter A. Lind, Dan I. Andersson

**Affiliations:** Department of Medical Biochemistry and Microbiology, Uppsala University, Uppsala, Sweden; University of Edinburgh, United Kingdom

## Abstract

We previously reported that the distribution of fitness effects for non-synonymous and synonymous mutations in *Salmonella typhimurium* ribosomal proteins S20 and L1 are similar, suggesting that fitness constraints are present at the level of mRNA. Here we explore the hypothesis that synonymous mutations confer their fitness-reducing effect by alterating the secondary structure of the mRNA. To this end, we constructed a set of synonymous substitutions in the *rpsT* gene, encoding ribosomal protein S20, that are located in predicted paired regions in the mRNA and measured their effect on bacterial fitness. Our results show that for 3/9 cases tested, the reduced fitness conferred by a synonymous mutation could be fully or partly restored by introducing a second synonymous substitution that restore base pairing in a mRNA stem. In addition, random mutations in predicted paired regions had larger fitness effects than those in unpaired regions. Finally, we did not observe any correlation between fitness effects of the synonymous mutations and their rarity. These results suggest that for ribosomal protein S20, the deleterious effects of synonymous mutations are not generally due to codon usage effects, but that mRNA secondary structure is a major fitness constraint.

## Introduction

Understanding the mechanistic basis of the fitness effects of mutations is of general interest for evolutionary biology, systems biology and for elucidating the genetic basis of complex diseases [1]. Traditionally, in light of the central dogma, the focus has been on the effect that changes in the amino acid sequence have on protein function or on well-defined regulatory elements outside coding regions. In recent years it has become increasingly clear that considering only effects on amino acid sequence is insufficient with the recognition of, for example, the importance of copy number variation, small RNAs and epigenetic modification [2], [3], [4], [5], [6].

The redundant nature of the genetic code is a clear example where selection could act in favor of a specific coding DNA sequence, encoding the same amino acid sequence as a less favored DNA sequence. The differential usage of specific synonymous codons between species of all the domains of life as well as between genes within a genome has long been recognized, but the causes of these codon usage biases are still unclear [7], [8], [9], [10]. Codon usage biases are influenced by the neutral processes of genetic drift and mutational biases as well as by selective effects on protein synthesis and cellular function [10], [11]. Several possible mechanisms for these fitness effects have been described including translation efficiency [7] and accuracy [12], [13] as well as tRNA sensitivity and depletion [14], [15], [16], [17], codon context [18], [19], mRNA structure and stability [9], [20], [21], [22], [23] and changes in protein tertiary structure [24].

Experimental studies of the causes of the fitness effects of synonymous mutations are challenging because the mechanistic effects are interconnected and typically direct tests of a particular hypothesis are not possible and only correlative results are obtained. The fact that the majority of synonymous substitutions has relatively small effects on fitness as well as on biochemical parameters, such as mRNA stability or translational efficiency, further complicates these studies. To overcome the limitations of the assays used, typically the effects of many synonymous mutations are measured simultaneously, which can lead to sequences that are very divergent to evolutionary optimized natural sequences [10], [17], [20].

We have previously described the fitness effects of single nucleotide substitutions in two ribosomal protein genes in *S. typhimurium* using a fitness assay sensitive enough to detect the small changes in fitness caused by single synonymous substitutions [21]. In that study a weak but significant correlation between fitness and predicted changes in mRNA secondary structure was found [21]. However, given the interconnected nature of the possible levels of selective constraints, mRNA structure could only explain part of the variation in fitness. A more direct test of the importance of the main selective constraint could be performed if intragenic compensatory mutations restoring mRNA structure could be found. We constructed a set of synonymous substitutions in *rpsT* (encoding ribosomal protein S20) that are located in paired regions in the mRNA to test the hypothesis that mRNA secondary structure is an important selective constraint. If correct, several predictions can be made: (i) we would expect mutations in stem regions to have larger fitness effects than random mutations, (ii) we would not expect larger fitness effects by introducing rarer codons and (iii), most importantly, we would expect that the fitness of a synonymous stem mutant is increased by introduction of a second synonymous substitution to restore the base pair in the stem of the mRNA.

## Materials and Methods

### Strains and Media

We used *Salmonella enterica* Var. Typhimurium LT2 (designated *S. typhimurium* in the text) and derivatives thereof in all experiments. Liquid media were LB or SOC for strain construction and M9 minimal medium supplemented with 0.2% (w/v) glucose for competition assays. LB agar was supplemented with kanamycin (50 mg/l) or chloramphenicol (30 mg/l) when appropriate for plasmid maintenance and selection.

### mRNA Secondary Structure Prediction

The secondary structure of the *rpsT* mRNA, including 20 bases upstream the start codon and the open reading frame, was predicted using mfold (mfold.rna.albany.edu) [25] using default parameters except that the maximum distance between paired bases was limited to 40.

### Construction of Mutants

Genomic DNA from a *S. typhimurium* strain containing the wild type *rpsT* gene with an adjacent kanamycin resistance marker [21] was isolated (Genomic tip 100/G, QIAGEN) and used as template DNA for PCR. For each desired mutation a primer was designed with a 40 bp homologous region upstream of the mutation and 5–6 bp downstream and which was used for a PCR (Phusion High Fidelity DNA polymerase, Finnzymes) with a reverse primer located dowstream of the kanamycin resistance cassette with homology to the wild type *S. typhimurium* chromosome. The amplified DNA fragments containing part of the *rpsT* gene with a base substitution, the kanamycin resistance cassette and downstream homologous region were purified (illustra™ GFX™ PCR DNA and Gel Band Purification Kit, GE Healthcare) for use in 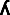 Red recombineering ([26]). The mutations were incorporated in the native *rpsT* gene in the *S. typhimurium* chromosome by introducing the linear DNA fragment into 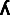 Red competent cells by electroporation and after shaking at 37°C for 3 hours the cells were plated on LA plates with kanamycin. Transformants were restreaked on LA kanamycin plates and mutants were found by DNA sequencing of the *rpsT* gene. Double mutants were also constructed using this protocol, but the template DNA for the PCR was isolated from a mutant containing the desired 3' mutation. Four wild type controls were constructed by an identical method with the only difference being that the sequencing did not reveal any mutations, i.e. failed mutagenesis. The *rpsT* genes containing the desired mutations were moved by P22 transduction into two strains carrying variants of the green fluorescent protein (*cfp*, *yfp*) that had been pre-adapted to the experimental environment used in the competition experiment, as previously described [27].

### Competition Experiments

Fitness of the mutants relative to the wild type were estimated by competition experiments using strains pre-adapted to the experimental conditions and encoding either the yellow (YFP) of cyan (CFP) variants of the green fluorescent proteins, as previously described [21]. Each mutation was introduced into both the CFP and YFP genetic background and competed against an isogenic wild type strain, also carrying the kanamycin cassette, with the other fluorescent marker. Overnight cultures of the mutant and wild type strains were mixed 1∶1 and grown in M9 glucose at 37°C with shaking (180 rpm). The initial ratio of mutant to wild type were measured by counting 100,000 cells for each competition using flow cytometry (BD FACS Aria) and the cultures were diluted 32-fold (5 generations per cycle) every 24 hours for up to six cycles and counted every cycle. Selection coefficients were estimated using the regression model s = [ln(R(t)/R(0))]/[t], as previously described [28], where R is the ratio of mutant to wild type and t the number of generations. The reported selection coefficients are the average of three to six independent competition experiments always including both the *yfp* and *cfp* genetic backgrounds. The small relative fitness cost of carrying the *cfp* marker compared with the *yfp* was measured during each round of competitions using four independently constructed wild type control strains and was subtracted from the selection coefficients for the mutants as previously described [27]. The Pearson correlation between the selection coefficients for the mutants in the *yfp* and *cfp* genetic backgrounds was 0.99 showing that the measured fitness costs are strongly dependent on the identity of the mutation.

## Results

### Secondary Structure Prediction and Construction of Mutants

**Figure 1:**
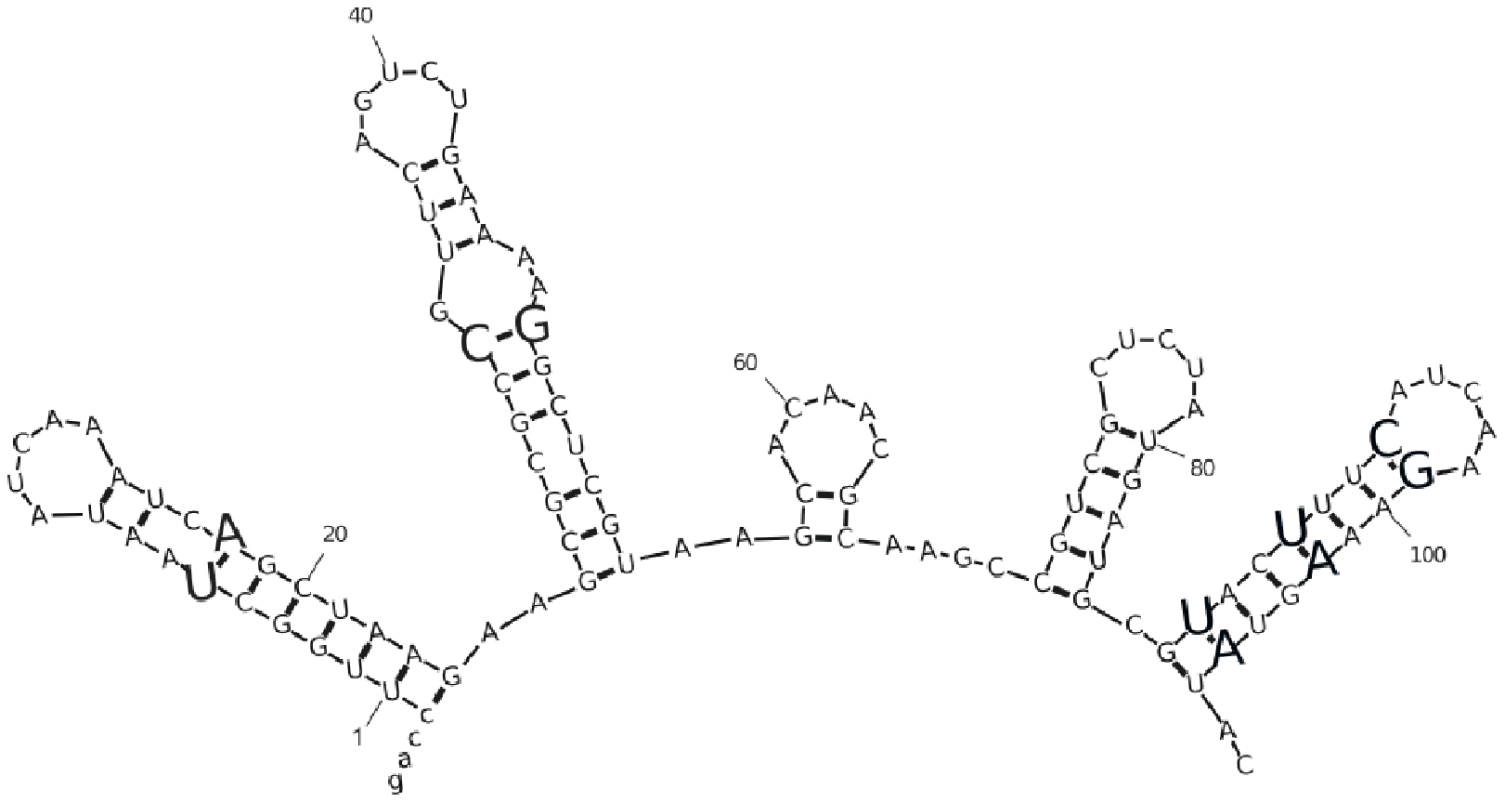
Predicted mRNA structure of part of *rpsT*. Numbering begins at TTG start codon and positions with where synonymous mutations have been introduced are shown in larger font.

We first made a computational secondary structure prediction for the *rpsT* mRNA using mfold (Fig. 1) [25]. Based on this prediction there were only ten sites in the gene that were in a paired region, a synonymous single step mutation was possible and there were also the rare oppurtunity of introducing complementary synonymous substitution restoring secondary structure (Fig. 1). In these ten sites, 18 single mutations were introduced into the native *rpsT* gene in the *S. typhimurium* chromosome using the lambda Red system for homologous recombination (Fig. 1, Table 1). These mutations were then combined to make 9 double mutations predicted to restore base pairing. Fitness was measured as the selection coefficient (s) of each mutant in a serial passage experiment in competition with an isogenic wild type strain. The ratio of mutant to wild type cells over time was determined by flow cytometric counting of single cells, where the wild type and mutant cells can be distinguished by expressing either the yellow (YFP) or cyan (CFP) variants of the green fluorescent protein [27].

**Table 1:** Summary of Single and Double Synonymous Mutations in *rpsT.*

Mutation	Selection coefficient	SEM	Amino acid	Native codon	Mutant codon	Rib freq native	Rib freq mutant	ln rib freq(mut/native	Native codon in gene	Mut codon in gene
T6A	−0.0112	0.0015	ALA	GCT	GCA	0.43	0.28	−0.43	12	6
A18T	−0.0186	0.0026	SER	TCA	TCT	0.06	0.38	1.85	1	2
T6A A18T	−0.0004	0.0017						1.42		
T6G	−0.0199	0.0012	ALA	GCT	GCG	0.43	0.18	−0.87	12	0
A18C	−0.0202	0.0010	SER	TCA	TCC	0.06	0.24	1.39	1	0
T6G A18C	−0.1336	0.0103						0.52		
T6C	−0.0186	0.0033	ALA	GCT	GCC	0.43	0.11	−1.36	12	1
A18G	−0.0146	0.0018	SER	TCA	TCG	0.06	0.02	−1.10	1	0
T6C A18G	−0.0140	0.0015						−2.46		
T87C	−0.0030	0.0007	ARG	CGT	CGC	0.67	0.31	−0.77	5	2
A105G	−0.0221	0.0017	VAL	GTA	GTG	0.21	0.14	−0.41	1	1
T87C A105G	−0.0251	0.0019						−1.18		
T87A	−0.0124	0.0016	ARG	CGT	CGA	0.67	0.01	−4.20	5	0
A105T	−0.0111	0.0008	VAL	GTA	GTT	0.21	0.53	0.93	1	1
T87A A105T	−0.0255	0.0016						−3.28		
T87G	−0.0047	0.0009	ARG	CGT	CGG	0.67	0.0063	−4.67	5	0
A105C	−0.0163	0.0031	VAL	GTA	GTC	0.21	0.12	−0.56	1	0
T87G A105C	−0.0233	0.0007						−5.23		
T90C	−0.0235	0.0032	THR	ACT	ACC	0.45	0.45	0.00	2	0
A102G	−0.0163	0.0029	LYS	AAA	AAG	0.71	0.29	−0.90	9	5
T90C A102G	−0.0223	0.0016						−0.90		
C93T	−0.0211	0.0018	PHE	TTC	TTT	0.73	0.27	−0.99	1	1
G99A	−0.0042	0.0013	LYS	AAG	AAA	0.29	0.71	0.90	5	9
C93T G99A	−0.0030	0.0011						−0.10		
C33T	−0.0215	0.0007	ALA	GCC	GCT	0.11	0.43	1.36	1	12
G48A	−0.0234	0.0023	LYS	AAG	AAA	0.29	0.71	0.90	5	9
C33T G48A	−0.0214	0.0024						2.26		

### Fitness Effects of Mutations

**Figure 2:**
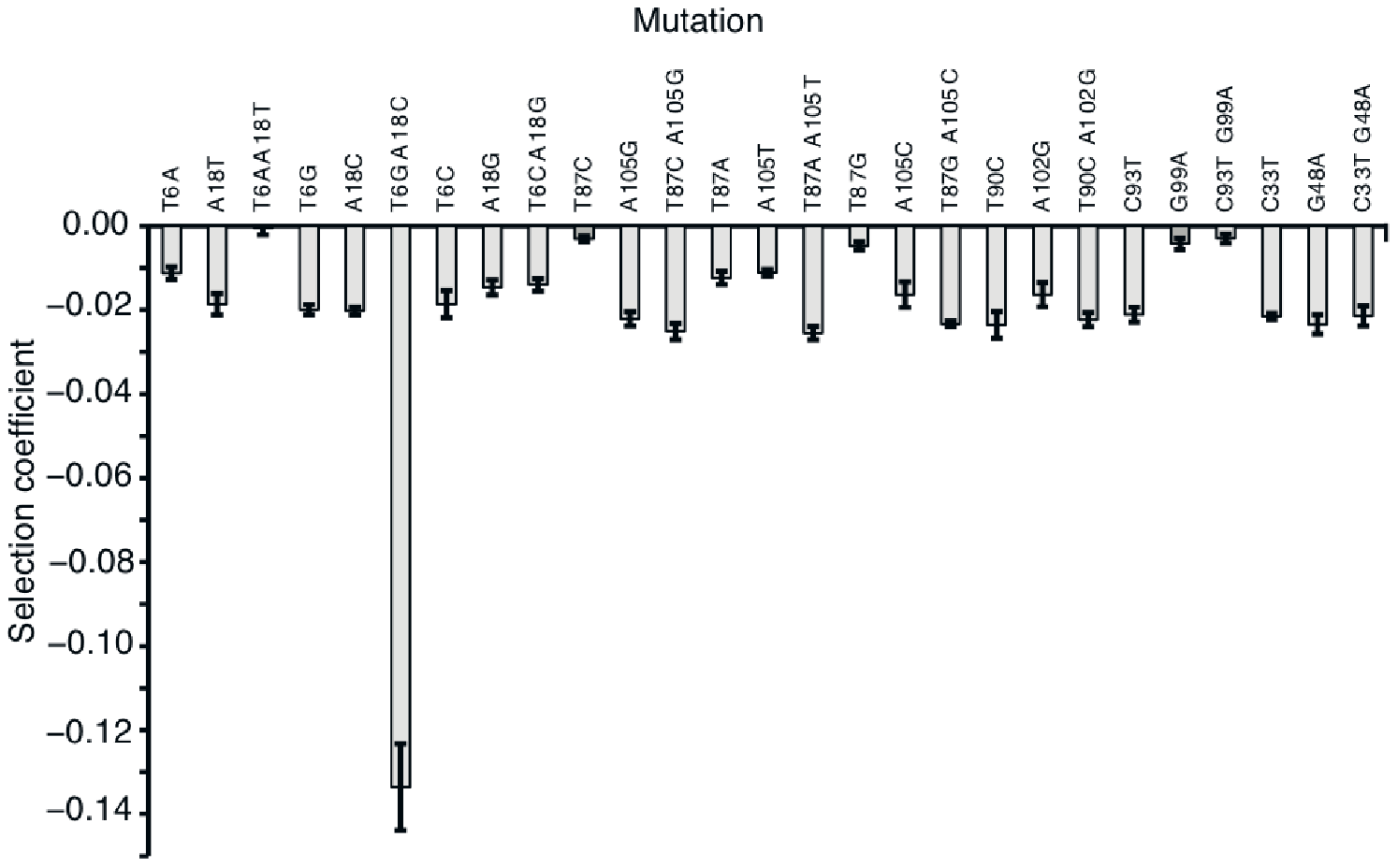
Fitness effects of synonymous mutations in *rpsT*. Selection coefficients were measured in competition with isogenic wild type strains; a selection coefficient of 0 is neutral and negative values are deleterious. Error bars represent SEM.

The majority (17/18) of the single synonymous substitutions reduced fitness more than the limit of detection of |s| = 0.003 with an average cost of s = −0.016 (Fig. 2, Table 1). This result confirms our previous findings and show that in this gene the frequency of neutral mutations is very low [21]. The double mutations reduced fitness in seven out of nine cases and in two cases (T6A–A18T and C93T–G99A) the fitness was restored to wild type levels with the introduction of a second substitution predicted to restore mRNA structure. In one case partial compensation was observed (T6C–A18G) and for two other double mutants (T90C–A102G and C33T–G48A) the fitness cost were similar to the single mutant with the largest effect, suggesting that mRNA structure was not restored. In three cases, all in positions 87 and 105, the selection coefficients of the double mutants were approximately additive and indicating that these bases do not interact in the mRNA secondary structure. For the T6G–A18C mutant the fitness cost was much larger (s = −0.134) than expected from the single mutants (T6C s = −0.020; A18C s = −0.020. This could be caused by an unfavorable stabilization of the mRNA structure by switching an AT to a GC base pair [9], [20], but this seems unlikely as the T6C–A18G mutant showed partial compensation. Another possibility is that the T6G–A18C mutation leads to a major change in mRNA structure as suggested by secondary structure prediction (Fig. S1).

### Codon Usage and Fitness Effects

**Figure 3:**
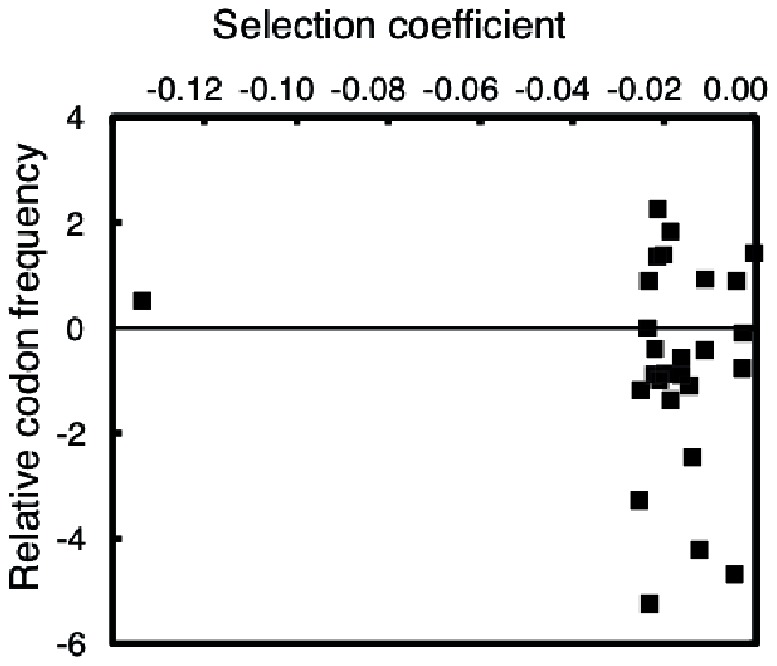
Changes in codon usage frequencies were not correlated with fitness costs of mutations. The frequencies of codons in *S. typhimurium* ribosomal protein genes were used to determine the ratio between the mutated and native codons. The relative codon frequency is the natural logarithm of this ratio.

Changes to codons more rarely used in *S. typhimurium* ribosomal protein genes were not significantly correlated with loss of fitness (Pearson correlation −0.09, Fig. 3) and no obvious connection was found when analyzing the intragenic changes in codon usage [19], but the number of codons in the *rpsT* gene (87 including start and stop codons) is too small for a proper statistical analysis. A minor role of codon usage is also supported by the mild fitness effect of introducing very rare arginine codons (CGA and CGG) compared to more commonly used codons (Table 1). In addition, we previously showed that replacement of *S. typhimurium* ribosomal protein genes with orthologues from other related species with large differences in codon usage typically had smaller fitness effects than those found in this study, again supporting a minor selective role for codon usage in these genes [27].

### Mutations in Paired Regions have Bigger Effect than Mutations in Unpaired Regions

The distribution of fitness effects for the 18 single synonymous substitutions in mRNA paired regions reported here can be compared to that of 21 random synonymous mutations in *rpsT* previously described [21]. As expected, if mRNA secondary structure is of major importance, the fitness costs would be significantly larger for the substitutions designed to be in paired regions compared to the random mutations (average random s = −0.009, average paired s = −0.016, two tailed t-test p = 0.047). However, this may be misleading as the mutations here are not independent (there are several in the same position) but if all 70 random synonymous and non-synonymous substitutions in *rpsT* previously reported [21] are divided into paired and unpaired regions in the predicted mRNA secondary structure there is a significantly higher cost for the paired ones (average paired s = −0.019, average unpaired s = −0.010, two tailed t-test p = 0.007). This suggests that for the *rpsT* gene mRNA structure is of primary importance, even more so than the effects of amino acid substitutions.

## Discussion

Our results shows that it is possible to restore fitness by introducing compensatory mutations based on restoration of base pairing in the mRNA of *rpsT* and support a major role for mRNA secondary structure in relation to the fitness effects of synonymous mutations. However, partly or fully restored fitness by predicted secondary structure restoration was only observed in three out of nine cases, which may seem like a minor success in predicting fitness effects. There are several reasons why we would not expect the fitness of the double mutant to return to wild type levels in many cases. First, the computational predictions of mRNA structure used here may not be able to predict a fully accurate secondary structure and we did not take into account that there are likely to be several different structures present in the cell. In addition the translational kinetics are likely to influence mRNA secondary structure and it is likely that not all structural elements are equally important for optimal function. Another simplification is the interchangability of the base pairs, so that no consideration is taken to the order or type of base pair, e.g. GC or AT. It is also likely that other effects do have a significant contribution to the fitness effects including tRNA usage, location in gene and RNAse sites.

The fitness effects of the synonymous mutations is likely to be caused by changes in mRNA stability or translation efficiency, which could result in lower levels of functional protein. In theory the experimental investigations into the mechanistic causes of the fitness effects are straightforward. Standard methods for measuring the levels of specific mRNAs and proteins are well established and it is also possible to determine the ribosomal occupancy of different regions of the mRNA. However, these methods do not have the sensitivity required to analyze the minor changes caused by single substitutions, which if directly correlated to fitness would be in the order of 1%, and the genetic approach used in this study was motivated by our failure to detect any changes in mRNA levels [21].

It is clear that over evolutionary timescales, synonymous sites are subject to fewer fitness constraints than non-synonymous sites as seen in genomic data, whereas we here find that the major constraints are on the mRNA level. We can also be confident that natural selection cannot be blind to this magnitude of fitness effects for ecologically relevant traits. It is possible that ribosomal protein genes may be different from the average gene because of their very high expression and direct connection to ribosome performance, which could translate minor mechanistic effects into substantial selective constraints and allow the optimization of expression to a higher degree than for genes with that are more influenced by stochastic variation of their mRNA levels. Strong selection for translational robustness, defined as the ability of proteins to fold properly despite mistranslation, is expected to result in the evolution of proteins that are extremely tolerant to missense mutations [29], which could explain the minor effects of most amino acid substitutions in ribosomal protein genes. The previously reported [27] mild fitness effects of replacing ribosomal proteins gene with orthologues from closely related species suggest that even though the majority of random substitutions are deleterious, the cumulative impact of many potentially neutrally fixed mutations conserves the important selective features on the mRNA level. Thus, the overrepresentation of synonymous substitutions in genomic data may reflect a larger neutral network for synonymous substitutions, so that there are many readily accessible alternative neutral mRNA structures even though most single substitutions are deleterious.

The scarcity of reports of effects of synonymous mutations in experimental studies is likely to be caused by their expected mild phenotype, so that in a mutation study under strong selection they are unlikely to be detected. For the same reason they are also less likely to turn up in experimental evolution studies where large effect mutations will be fixed first. So, although synonymous mutations are unlikely to contribute to evolutionary innovations they will be important under weak selection to optimize expression levels and may have a larger role to play than their use as assumed neutrally evolving sites.

## Supporting Information

Figure S1 **The mutations T6G and A18C causes a large predicted change in the secondary structure of the first stem loop of the** ***rpsT*** **mRNA.**
(PDF)
