# Supplementary figures and images for: Fitness Costs of Synonymous Mutations in the rpsT Gene Can Be Compensated by Restoring mRNA Base Pairing
Source: PLoS One. 2013 May 15;8(5):e63373. doi: 10.1371/journal.pone.0063373 (PMC3655191; doi:10.1371/journal.pone.0063373)

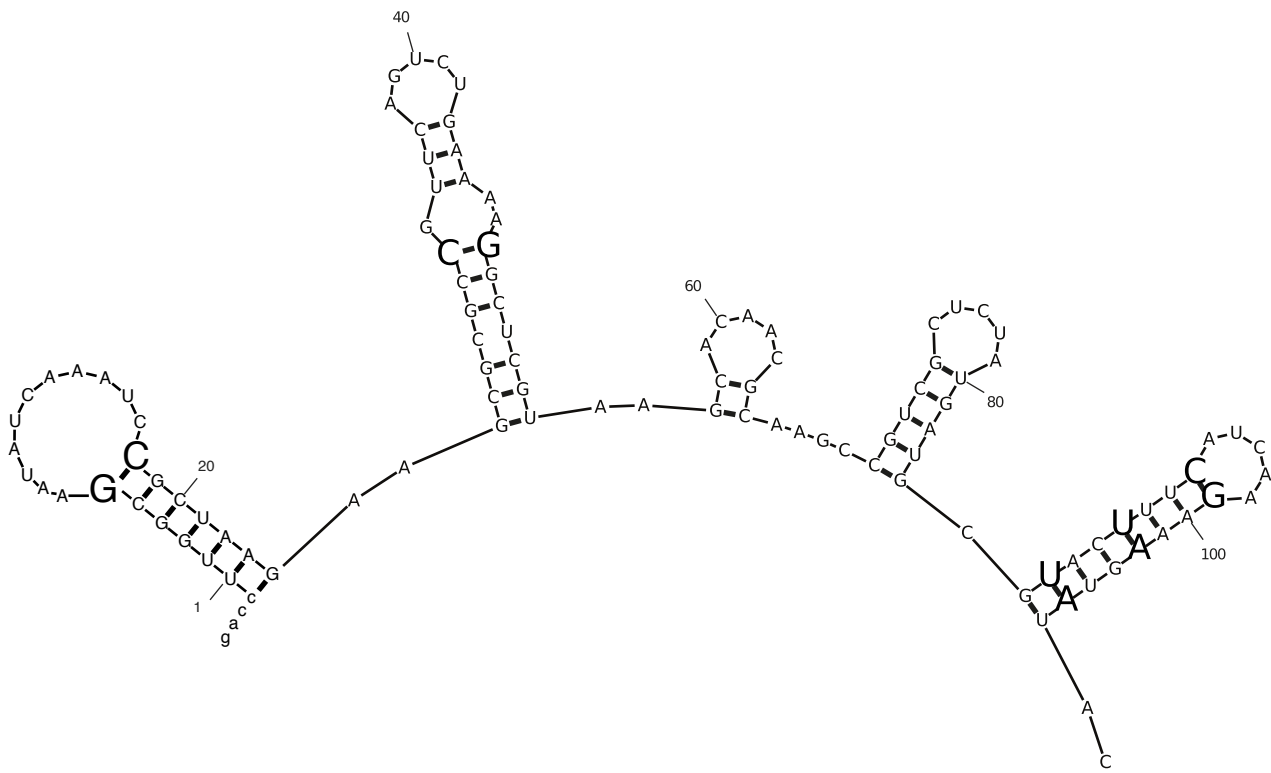

Supplement: Figure S1 — The mutations T6G and A18C causes a large predicted change in the secondary structure of the first stem loop of the rpsT mRNA. (PDF) [file pone.0063373.s001.pdf]
